# Visual requirement for Chinese reading with normal vision

**DOI:** 10.1002/brb3.1216

**Published:** 2019-02-21

**Authors:** Chen‐Xiao Wang, Na Lin, Ying‐Xuan Guo

**Affiliations:** ^1^ School of Optometry and Ophthalmology and Eye Hospital Wenzhou Medical University Wenzhou Zhejiang China

**Keywords:** acuity reserve, rapid serial visual presentation, reading performance, reading speed, visual performance

## Abstract

**Objectives:**

Reading performance has been considered as an effective functional endpoint for low vision. Contrary to many extensive studies for reading performance in English, there are few systematic studies for Chinese reading.

**Methods:**

In the present study, the reading performance of 30 normally sighted Chinese college students was systematically investigated. All participants passed the equivalent test of Cambridge ESOL PET in China. The reading speeds for Chinese and English text at a variety of text sizes were measured with rapid serial visual presentation (RSVP). The threshold acuities for Chinese characters and English letters were measured. Maximum reading speed, critical font size, and critical acuity reserve were derived according to the individual's reading speed curve.

**Results:**

The maximum reading speed for Chinese characters was 259.5 ± 38.2 characters/min, which was significantly faster than that for English letters (135.7 ± 18.5 words/min, *p = *2.8 × 10^‐18^). The critical font size for Chinese characters was larger than that for English letters (24.2 ± 2.8 arcmin vs. 20.7 ± 1.0 arcmin, *p* = 1.6 × 10^‐7^). Interestingly, the critical acuity reserve was similar for these two languages (3.4 ± 0.4 for Chinese and 3.4 ± 0.2 for English, *p* = 0.4).

**Conclusion:**

The present study provides the first step for establishing visual functional endpoints for Chinese reading. Our findings pose rigorous constrains on present theories in language information processing and brain plasticity.

## 1 INTRODUCTION

Reading is a fundamental skill that involves a series of processes of visual perception and cognition. It is also a common daily visual task for almost everyone. Reading proficiency requires normal optometric and ophthalmological pathways. Thus, functional deficits or pathology in central and peripheral vision could significantly affect reading performance. Reading performance has been considered as an effective functional endpoint for low vision (Legge, Rubin, Pelli, & Schleske, 1985; Whittaker & Lovie‐Kitchin, 1993). Reading difficulty has been suggested as a therapy priority for low‐vision patients (Owsley, McGwin, Lee, Wasserman, & Searcey, 2009; Willis & Ramulu, 2006; Zebardast, Friedman, & Vitale, 2017). Since the aged population, especially the literate aged population, is increasing in China, low vision caused by age‐related chronic disease is becoming a tremendous challenge to society (He, Wang, & Huang, 2017; Lou, Wang, Xu, Ye, & Ye, 2017; Wang, Yan, Muller, Keel, & He, 2017). Therefore, an investigation in reading is of theoretical and clinical importance.

Reading performance is typically quantified as reading speed in unit of words per minute (wpm). Text properties, such as font size and contrast, that affect reading speed has been extensively studied (Brussee, Berg, Nispen, & Rens, 2017; Legge, Rubin, & Luebker, 1987; Legge, Rubin et al., 1985). As the font size increases from the threshold level, reading speed rapidly increases, and the maximum speed plateaus at large print sizes (Legge, Rubin et al., 1985; Mansfield, Legge, & Bane, 1996). Shi et al. (2010) found that reading speed for children with low vision is not correlated with their visual acuity. After realizing that text size threshold (visual acuity in other words) and print size alone does not predict reading speed, Whittaker and Lovie‐Kitchin (1993) proposed the “acuity reserve” to estimate the reading requirement for low‐vision patients. Acuity reserve is the ratio between the font size used in reading and the acuity threshold. Hence, the acuity threshold for Chinese characters has to be measured. The assessment of critical acuity reserve for reading could allow a clinician to identify visual impediments to reading, and individualize interventions for low‐vision patients.

Although there have been many extensive studies in the reading performance of native English speakers, there is a lack of systematic studies in reading performance of native Chinese speakers. Efforts have been given in studying Chinese reading performance under different presentation paradigms, such as leading versus rapid serial visual presentation (RSVP) (Shieh, Hsu, & Lin, 2005), and word by word versus sentence by sentence (Chen & Chien, 2005). Scholars have also tried to investigate the effect of font style or font size (Chan & Ng, 2012; Legge, 2016), and perceptual span (Yan, Zhou, Shu, & Kliegl, 2015) on reading. However, in these studies, merely few of the font sizes were used. The limited size condition makes it difficult to derive the full relationship between reading performance and size. Given the fact that the font sizes used in these studies were above the threshold, their result could not provide any information on the critical font size for reading performance. Moreover, there were even fewer studies in the acuity reserve for Chinese reading, which is most critical in vision quality assessment in clinical practice. Ma, Qu, and Xu (2004) measured the acuity reserve for Chinese readers. However, they only used four different font sizes in their study, which may not be enough to capture the entire reading speed curve, and this could substantially bias the estimated critical font size and acuity reserve.

The necessary step to calculate acuity reserve is to measure threshold acuity. Chinese characters are significantly different from English letters in spatial structure. Researchers have found that the threshold acuity for Chinese characters was higher than that for Snellen E (Zhang, Zhang, Xue, Liu, & Yu, 2007, 2009). Moreover, the spatial structure of Chinese characters varies from each other in a wide range of spatial complexity, such as from one stroke to more than 60 strokes. In order to estimate the acuity reserve for Chinese characters, the threshold acuity for Chinese characters should be precisely measured.

The present study aimed to systematically investigate the performance for Chinese reading by measuring reading speed as a function of font size in a group of Chinese college students. A Chinese visual acuity chart with the consideration of the spatial spectrum of Chinese characters was first designed, and this was used to measure threshold acuity. In order to make the result comparable, an English visual acuity chart with a similar procedure was also established. The reading speed curves for the Chinese and English language were measured. The acuity reserve and critical acuity reserve for Chinese and English were derived and compared.

## 2 METHODS

### 2.1 Participants

Thirty graduate students from Wenzhou Medical University voluntarily attended the present study (age: 25.1 ± 3.2 years old, mean ± *SD*). All of them underwent a detailed ophthalmological and optometric examination performed by the first author, CW. These students did not show any sign of any eye problem/disease. Five subjects were emmetropic, while 25 subjects had refractive errors. The refractive status in these subjects ranged from 0 to −9.00 spherical diopter (−3.3 ± 2.45 D) and 0 to −1.25 cylindrical diopter (−0.40 ± 0.45 D), with an accommodation amplitude between 5.75 D and 9.75 D (7.37 ± 0.91 D). All participants were native Chinese, and had a visual acuity or best corrected visual acuity of at least 20/20. Furthermore, all participants passed the College English Test 6 (CET 6), which is considered to be an equivalent of Cambridge ESOL PET (Tang, Pritchard, & Shi, 2012) in China. Moreover, all participants were naïve to the purpose of the experiment. A written informed consent was obtained before the experiment was started. The study adhered to the tenets of the Declaration of Helsinki, and was approved by the Ethics Committee of Wenzhou Medical University.

### 2.2 Apparatus

All stimuli presentations and data collection were performed using a PC computer that ran customized programs. The computer had a recording and speaking system. Stimuli were presented on a 19‐inch monitor with a resolution of 1,280 × 1,024 pixels. The contrast of the stimuli was 98.4% (black stimuli on white background). During the experiment, fluorescent lights with a desk luminance of 180 lux provided ambient illumination.

### 2.3 Stimuli

#### 2.3.1 Stimuli for measuring threshold acuity

**Figure 1:**
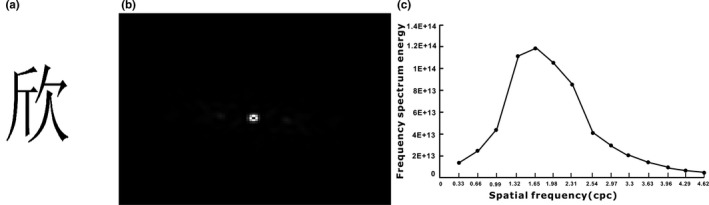
Spatial property of visual stimuli for Chinese characters. A Chinese character selected for testing visual acuity (a); its power distribution on the two‐dimensional spatial frequency domain (b), and the mean radial frequency spectrum for the selected Chinese character set and English letter set (c). The error bar represents the standard deviation, cpc represents the cycles per character, and cpl represents the cycles per letter

In order to accurately estimate the acuity reserve, a set of Chinese characters was first carefully chosen, and this was used to measure the threshold acuity for Chinese reading. Although there are up to 80,000 Chinese characters, merely 2,500 of these are commonly used. The most frequently used Chinese characters have eight strokes (Institute of linguistics CASS, 2004). Thus, 309 of the most commonly used Chinese characters with eight strokes were selected for further processing (Chinese Characters Division, National Committee on Language and Literature, 1988). The character images were generated using Adobe Photoshop 7.1 (Adobe Systems Incorporated, San Jose, CA, USA). A black character with size of 300 × 300 pixels was centered on a white background of 900 × 900 pixels (Figure 1a). Each character image was Fourier‐transformed into a two‐dimensional spatial frequency domain using our Matlab (Mathworks, Inc.) program (Figure 1b) (Wang et al., 2008). Then, the spatial spectrum of each character was radially averaged, resulting in a one‐dimensional radial frequency spectrum (Figure 1c). All eight‐stroke characters were classified according to the Euclidean distance of the one‐dimensional spectrum. In the largest class of characters, those that had more even stroke distributions across the space were selected. The Chinese character set that resulted from this classification was 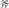, 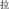, 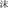, 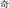, 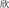, 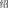, 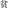, 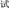, 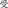 and 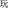. As most English reading materials are printed in lowercase, similar procedures were also used to select a group of lowercase English letters with similar spatial frequency for English visual targets. These letters were f, y, c, v, z, r, t, j, l, and i.

**Figure 2:**
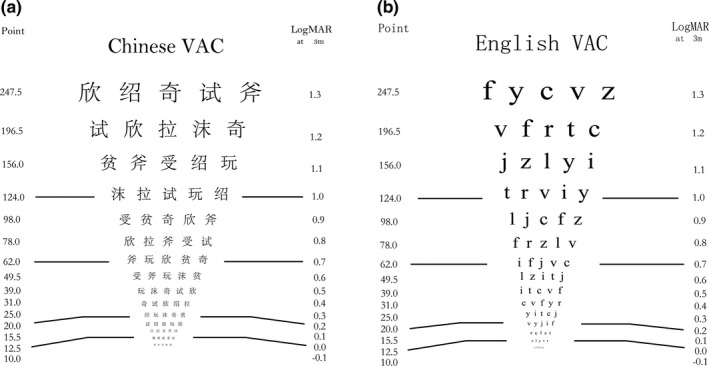
The self‐designed Chinese (a) and English (b) visual acuity chart. The Chinese characters include 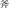, 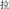, 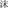, 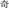, 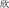, 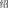, 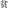, 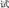, 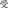 and 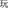. The English letters include f, y, c, v, z, r, t, j, l, and i. The characters or letters (as visual targets) had 15 different sizes arranged in descending order from 1.3 LogMAR to −0.1 LogMAR, with increments of 0.1 log unit between two nearby rows

These selected characters or letters were used to generate separate Chinese and English acuity charts (Figure 2). The font style was Song for Chinese visual targets and Times New Roman for English visual targets. The font size for English letters was defined as x‐height. The font style of Song is a fixed‐–width font style. The size for Chinese characters was defined as the height of the minimum bounding rectangle to enclose all characters in the chart (that are center‐aligned). According to “Recommended Standard Procedures for the Clinical Measurement and Specification of Visual Acuity” (Committee on Vision, 1980), the size of 1 logMAR optotyope should be 50 arcmin. Hence, we adopted this idea, and defined that English and Chinese characters subtend 50 arcmin correspond to 1 logMAR in the present customized visual acuity chart. The charts had 15 rows, and each row had five randomly selected characters or letters of the same size. From top to bottom, the size of each row decreased from 1.3 to −0.1 logMAR, with decrements of 0.1 logMAR. In order to test the threshold acuity, these charts were presented on a computer screen. Due to the size limit of the screen, only part of the chart (approximately three rows) was presented once. The experimenter scrolled the chart left and right or up and down, according to the response of the participant.

#### 2.3.2 Stimuli for measuring reading speed

The RSVP paradigm was used to measure reading speed. The words or characters of a sentence were presented sequentially, one at a time, at the same location on the display for a fixed duration of exposure. There was no blank frame (interstimulus interval) between each pair of words. The exposure duration was determined by the presenting speed: duration = 60 (seconds per min)/speed (wpm or cpm). Chinese sentences were presented character‐by‐character while English sentences were presented word‐by‐word. It is because, in Chinese, characters were the minimum semantic unit for recognition (Xu, 2001). On the other hand, words were the basic unit for recognition in the case of English (Jackson & Amvela, 2000). Twenty articles from Chinese high‐school text books (Grade 10 level; font: Song) or 20 English articles from the English Broadcasting Program of Voice of America (equivalent to Grade 10 level; font: Times New Roman) were selected as reading materials. The number of characters or words in each article ranged within 189–245 (213 ± 15) Chinese characters or 142–204 (167 ± 20) English words, respectively. Articles for the reading test were randomly selected, and each article was only used once. Generally speaking, the ratio of Chinese character count to English word count was roughly 1.6:1.0 to convey the same amount of information (Taylor & Taylor, 1983). Ten font sizes were used, which were linearly sampled in log space from 12.1 to 100.0 arcmin, with an increment of 0.1 log unit between sizes. All participants viewed the stimuli binocularly at a distance of 40 cm, and read aloud.

### 2.4 Procedure

#### 2.4.1 Measurement of threshold acuity

The customized Chinese or English visual acuity charts were presented on the screen. The spacing between characters/letters was the same as the width of the character/letter. The participants viewed the screen binocularly at 3 meters. Starting with the largest stimuli, the participants were asked to read the visual targets until at least three targets in a row were not readable (Raasch, Bailey, & Bullimore, 1998). The total number of readable visual targets per participant was used to calculate the threshold acuity in logMAR using the following formula, based on previous reports (Carkeet, 2001; Carkeet, Lee, Kerr, & Keung, 2001): logMAR = 1.4–0.02 × total number of readable visual targets. Then, the threshold acuity in arcmin for reading was converted from the acuity in arcmin with the equation 10^logMAR^ × 5.

#### 2.4.2 Measurement of reading speed

In order to measure the reading speed, all subjects were requested to read the reading materials aloud under different presenting speeds and different text sizes. For each text size, the participants read the article, one paragraph at a time, at different presenting speeds from slow to rapid. The time spent for reading each paragraph (reading time) and percentage correct of reading were recorded by the experimenter. The experimenter adjusted the presentation rate until the accuracy of the participant reached 80% to 90%. After the adjustment of presentation rate, the participants continued to read next paragraph or the first paragraph of a new article if they reached the end of the article. Generally, each paragraph contains 30–40 characters or words and at each print size, the participants read 4–10 paragraphs in total. The method of adjustment similar to a reference was used (Legge, Rubin et al., 1985).

When the correct reading rate reached 80%‐90%, the presenting speed was recorded (Legge, Pelli, Rubin, & Schleske, 1985). The reading speed was calculated using the following formula (Legge, Pelli et al., 1985): reading speed (wpm or cpm) = correct reading rate (%) × total number of words or characters × 60/reading time (in second).

The reading speed for Chinese and English was measured as functions of font size. A two‐limbed model was fit to the data to derive the maximum reading speed and critical font size for each participant (Legge, Pelli et al., 1985). Acuity reserve (R_A_) was used to describe the ratio of font sizes over the visual acuity threshold for reading materials through the following formula: R_A_ = S_P_/S_T_; where S_P _was the font size of the reading material printed for the reader, and S_T_ was the acuity threshold expressed as the font size (Whittaker & Lovie‐Kitchin, 1993).

### 2.5 Data analysis

We used customized programs written in Matlab to perform image filtering and other image processing in acuity chart design. To derive the maximum reading speed and critical font size for each participant and language (Legge, Pelli et al., 1985), a two‐limbed model was used and fit to the reading speed curve. The fitting procedure was done with a customized program in Matlab and via the method of least squares.

IBM SPSS statistics 24 was used for performing the cluster analysis on the candidates of Chinese characters or English letters. Paired *t *test was performed in IBM SPSS statistics 24 to compare the difference in reading performance between Chinese and English.

In Results, we report the reading performance of native Chinese speakers when reading Chinese (CC) and when reading English (CE). In Discussion, we compared what we have found in current study to the reading performance of native English speakers when reading English (EE) reported in the literature. In the following, we use the acronyms “CC”, “CE” and “EE” to indicate different conditions respectively.

## 3 RESULTS

### 3.1 Threshold acuity for Chinese characters and English letters

In order to estimate the acuity reserve, the threshold acuity for characters or letters should be first measured. Using carefully designed visual acuity charts, the threshold acuities for Chinese and English characters were measured. The averaged threshold acuities of these 30 subjects were 7.1 ± 0.9 arcmin (mean ± *SD*) and 6.1 ± 0.6 arcmin for Chinese and English, respectively. The threshold acuity for Chinese characters was significantly higher than that for English letters for our participants (paired *t *test, *t *(29) = 7.6, *p* = 2.0 × 10^−8^).

### 3.2 Critical font size and maximum speed in reading Chinese and English

**Figure 3:**
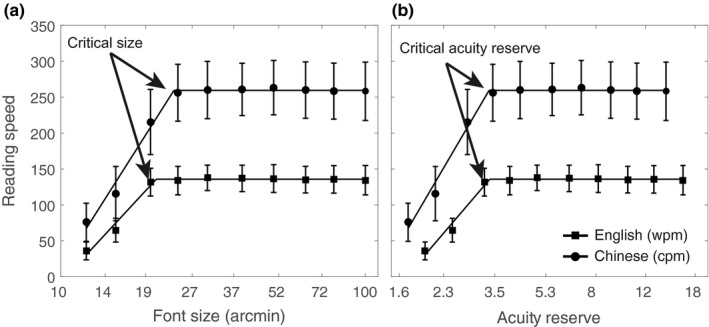
(a) The reading speed of Chinese and English as a function of font size. (b) The reading speed of Chinese and English as a function of acuity reserve. The critical font size and critical acuity reserve were obtained with the least square curve fitting method using a two‐limbed function (Legge, Pelli et al., 1985), which are indicated with arrows. The filled squares and circles represent the data for English and Chinese. The lines represent the best model fits. The error bar represents the standard deviation. The units of reading speed used for Chinese and English were cpm and wpm, respectively

The reading speed for CC and CE were plotted as functions of font size (Figure 3a). The reading speed curves of these two languages exhibited similar patterns. These both increased with the increase in font size in reading materials, and the maximum reading speed reached when the critical font size was met. The maximum reading speed remained unchanged during the reading test with font sizes larger than the critical font size. The maximum reading speed and critical font size were derived with a best‐fitting two‐limbed model for each participant and language (Legge, Pelli et al., 1985)(see Method). The critical font size for CC was 24.2 ± 2.8 arcmin (*n* = 30), with a maximum reading speed of 259.5 ± 38.2 characters/min (cpm). The critical font size for CE was 20.7 ± 1.0 arcmin, with a maximum reading speed of 135.7 ± 18.5 words/min (wpm). The critical font size for CE was larger than that for CE (paired *t *test, *t *(29) = 6.9, *p* = 1.6 × 10^−7^). The maximum reading speed for CC were significantly faster than that for CE (paired *t *test, *t *(29) = 19.6, *p* = 2.8 × 10^−18^).

### 3.3 Critical Acuity Reserve

The acuity reserve (R_A_) was calculated for the reading data of both CC and CE. In Figure 3b, the reading speed was replotted as functions of the acuity reserve for both Chinese and English. Reading speed increased with the increase in acuity reserve. The maximum reading speed remained unchanged when the acuity reserve increased to certain levels. The critical acuity reserve is a minimal acuity reserve when the reading speed reached a maximum value. Interestingly, the critical acuity reserves were 3.4 ± 0.4 and 3.4 ± 0.2 for CC and CE, respectively, and the difference between these was not statistically significant (paired *t *test, *t *(29) = 0.8, *p* = 0.4).

The critical font size and critical acuity reserve were obtained from the best‐fitting two‐limbed function (see Method), which were indicated with arrows. The filled squares and circles represent the data for CE and CC. The lines represent the best model. The error bar represents the standard deviation. The units used for reading speed for CC and CE were cpm and wpm, respectively.

## 4 DISCUSSION

In the present study, the threshold acuity for both Chinese characters and English letters were measured using carefully designed visual acuity charts. The mean threshold acuity in the group of Chinese college students was 7.1 ± 0.9 arcmin for Chinese and 6.1 ± 0.6 arcmin for English, respectively. Then, reading speed was measured at various font sizes, and the maximum reading speed, critical font size, and critical acuity reserve were derived. The maximum reading speed for CC and CE was 259.5 ± 38.2 cpm and 135.7 ± 18.5 wpm, respectively. In addition, the critical font size for CC and CE was 24.2 ± 2.8 arcmin and 20.7 ± 1.0 arcmin, respectively, and the critical acuity reserve for CC and CE was 3.4 ± 0.4 and 3.4 ± 0.2, respectively. It was found that CC condition showed a higher maximum reading speed, and required a higher threshold acuity and critical font size than CE. However, the critical acuity reserves for CC and CE were virtually the same.

The spatial complexity of Chinese characters brings a lot of troubles in designing an appropriate optotype for Chinese (Zhang, Zhang, Xue, Liu, & Yu, 2007). The approach of the present study was to select characters that have the most similar spatial structure among the most frequently used character set. The resulting optotypes were very similar to the Chinese character group 3 in a reference (Zhang, Zhang, Xue, Liu, & Yu, 2009), which also had 8–9 strokes per character. In fact, that study revealed that the visual acuity versus optical defocus functions of the character group 3 and Snellen E had similar slopes, and differed only by a vertical shift. The finding actually supported a simple linkage between the visual acuity of the Chinese acuity chart in the present study and the conventional Snellen acuity.

Lower case letters were used in the acuity chart of the present study, because most of the English reading materials were printed in lowercase. The visual acuity of the present participants using the standard chart was 20/20 or less, which corresponds to 5 arcmin of print size, while the lower case visual acuity was 6.1 arcmin. The acuity measured in the present study only serves as a basic visual limitation for English and Chinese. The acuity reserve reported in current study would be more accurate as the print size is divided by the lower case visual acuity.

All subjects were measured using the specially designed visual acuity charts, and it was found that the visual acuity threshold for Chinese characters (7.1 ± 0.9 arcmin) was significantly higher than that for English letters (6.1 ± 0.6 arcmin). The difference in threshold acuity between these two languages was possibly due to the difference in spatial structure between Chinese characters and English letters. The median number of strokes for most common Chinese characters is eight. However, English letters only have 2–3 equivalent strokes. In the object frequency domain, the spectrum for Chinese characters peaked at a higher frequency than that for English letters, and Chinese characters had a relatively higher frequency component than English letters. Therefore, in the spatial frequency domain, in order to achieve a similar spectrum, a larger size is required for Chinese characters, when compared with English letters. Guo (1999) reported that there is a positive correlation between the number of strokes and total Fourier frequency spectrum energy. The number of strokes for visual target is one of the important factors for determining the visual acuity threshold (Guo, 1999). Therefore, the visual acuity threshold in Chinese is higher than that in English.

It was also found that the critical font size for CC is larger than that for CE. The maximum reading speed for CC is much lower to the maximum reading speed for EE reported in the literature (Legge, Pelli et al., 1985). This is very possible due to the same reasons of the difference in spatial structure between Chinese characters and English letters. He, Baek, and Legge (2018) compared Korean and English RSVP reading speed in bilingual Korean readers and found larger critical print size when reading the more complex Korean text. They also contribute the finding to the additional within‐character crowding from more complex orthography.

The maximum reading speed for CE was much lower than that for EE reported in the literature (Legge, Pelli et al., 1985), given that all subjects learned English for more than 10 years. As a second language, grade 10 level English would still be difficult for the present participants. It is possible that this is one of the reasons that caused the slower reading speed for English. It was also possible due to the extra complexity in spatial structure in Chinese characters, which requires more processing resources of the visual cortex.

Interestingly, it was found that the critical acuity reserves for CC and CE were the same. It was postulated that the critical acuity reserve, which reflects the functional constraint set by our brain, limited the reading performance for CE. It is possible that native Chinese speakers process the second language using same mechanism they used for processing their native language. As native Chinese speakers, their brain has been shaped to process Chinese characters during the critical development period, and the system could be less efficient when processing English letters. Whether this functional constraint can be removed or processing efficiency can be improved through training remain as interesting questions for future studies.

In summary, reading speed was systematically measured under different sizes in the Chinese and English language in a group of Chinese college students. The critical size, maximum reading speed, and critical acuity reserve were derived and compared across conditions. It was found that for normal Chinese readers, reading speed in the Chinese language was faster than that in the English language. Furthermore, the participants in the present study required a higher visual acuity threshold and larger critical font size in reading Chinese characters, when compared to English letters. This finding reflects the structural difference between Chinese characters and English letters. The maximum reading speed for CE was lower than that reported in other studies, suggesting that the difference in visual information processing between native and second languages. We also found the critical acuity reserve was similar between CC and CE, which posed a rigorous constrain on current theories in visual information processing and brain plasticity. The present study provides a first step in investigating the reading requirement for Chinese readers with normal and low vision.

## CONFLICT OF INTERESTS

There is NO conflict of interest. The funders had no role in study design, data collection and analysis, decision to publish, or preparation of the manuscript.

## AUTHOR CONTRIBUTIONS

Chenxiao Wang conceived the experiments. Na Lin performed the experiments. Yingxuan Guo analyzed the data. Chenxiao Wang, Na Lin and Yingxuan Guo interpreted the data and wrote the manuscript. All authors reviewed the manuscript.
